# Sensory Processing Sensitivity and Compassion Satisfaction as Risk/Protective Factors from Burnout and Compassion Fatigue in Healthcare and Education Professionals

**DOI:** 10.3390/ijerph18020611

**Published:** 2021-01-12

**Authors:** Manuela Pérez-Chacón, Antonio Chacón, Mercedes Borda-Mas, María Luisa Avargues-Navarro

**Affiliations:** 1 International Doctoral School of the University of Seville, University of Seville, 41013 Seville, Spain; manpercha@alum.us.es (M.P.-C.); antpercha2@alum.us.es (A.C.); 2 Department of Personality, Assessment, and Psychological Treatment, University of Seville, 41018 Seville, Spain

**Keywords:** burnout, compassion fatigue, compassion satisfaction, sensory processing sensitivity, healthcare workers, educators, risk/protective factors, COVID-19

## Abstract

The study analyzes sensory processing sensitivity and the compassion satisfaction as risk/protective factors against burnout and compassion fatigue, during the first period of the COVID-19 health emergency. A sample of 1566 Spanish adult healthcare (n = 694) and education (n = 872) professionals was evaluated. An ad hoc questionnaire for sociodemographic data, and the highly sensitive person scale (HSPS), Maslach burnout inventory (MBI) and professional quality of life scale (ProQOL-vIV) were administered. Burnout and compassion fatigue were observed in the healthcare and education professionals, where personal realization and depersonalization were higher in healthcare and compassion fatigue in education. The protective role of compassion satisfaction was confirmed, as was sensory processing sensitivity as a risk factor, except for its low sensory threshold dimension, which positively influenced personal realization. The findings of this study demonstrate the presence of burnout and compassion fatigue in healthcare and education professionals, displaying compassion fatigue as an emerging psychosocial risk in education, which was made more severe under the conditions of study, which is at the beginning of the COVID-19 pandemic. The importance of incorporating adequate management strategies for high sensitivity, empathy and compassion satisfaction in prevention programs is emphasized.

## 1. Introduction

In recent decades, studies have reported that healthcare and education professionals are the groups most at risk of developing burnout because of their work in social service. In healthcare, professional activity is very emotionally demanding [1] due to its strong involvement in a job concentrating on human suffering. This is due to the emotional expectations generated and the need to constantly manage emotions adequately. The progressive state of stress experienced becomes a risk factor for developing burnout, depending on the working conditions and personal characteristics of the professionals [2].

Professional activity in education is also very physically and emotionally demanding. Today, the work of education professionals goes beyond teaching a subject matter. There is also a relationship in which they help with problems and concerns not directly related to education but also other areas (e.g., family and peers), and give attention to those students with special education needs (e.g., psychological, physical or sensory disability and/or behavior disorders, as well as highly gifted students) or in disadvantaged economic, sociocultural or health situations. Therefore, education involves an excessive emotional demand in the form of assistance, converting it in a risk factor for development of burnout [3].

In addition to burnout, recent studies on the quality of life of professionals devoted to helping others have referred to the concept of compassion fatigue. Compassion fatigue arises suddenly as the result of investing high levels of energy and compassion, or empathic care of others during an extended period of time. It is, therefore, not surprising that this syndrome has been widely studied in teaching [4] and healthcare [5].

Compassion fatigue has been described as a psychological state of anxiety or physical and mental distress associated with the stress of helping [6]. Not acting in time can affect one’s professional life by making it hard to care for patients/clients, and when empathy is not as strong, can evolve into burnout. Some authors have suggested that compassion fatigue is a syndrome related to emotional exhaustion, a basic component of burnout in labor [2], as a consequence of being exposed and absorbing the suffering of the patient/client for an extended period along with the professional’s own empathic predisposition.

The high demand of performing service work in healthcare and education can also be of benefit, as it is compensated by the satisfaction of contributing one’s professional and human capacity to helping and social service. This benefit, called compassion satisfaction [6], becomes a protective factor against developing burnout and compassion fatigue by increasing the capacity to support the emotional cost of concern for patients/clients [7]. In addition, as the choice of professions in healthcare and teaching tends to be vocational, intrinsic motivation predisposes to greater satisfaction from helping and caring for others. Some authors [8] have found a direct relationship between burnout and compassion fatigue and the opposite with compassion satisfaction [1]. 

Healthcare and education professionals who suffer from these syndromes show differences in personality variables when interacting with the persons they care for or provide service to. Several personal variables have been studied with regard to burnout and compassion fatigue [9]. These variables (e.g., personality, problems relating to and understanding patients/clients and/or coping strategies) could explain individual differences with respect to susceptibility to syndromes such as burnout or compassion fatigue.

Along this line, numerous studies in recent decades have related stress with personal risk and protection variables. Thus, a personality type characterized, among other factors, by the cognitive ability to perceive what the other person would feel in that situation is highly sensitive. Such people have sensory processing sensitivity, a trait which is related to feeling stressed by new or intense environmental or social stimuli. This trait represents a stronger awareness of sensory stimulation, inhibition of behavior and deeper cognitive awareness of environmental stimuli, leading to stronger emotional and physiological reactivity [10]. 

Although high sensitivity has been studied in recent years, its relationship to burnout or compassion fatigue has not received as much attention. However, a relationship has been found between highly sensitive individuals and reactivity to stress in traumatic situations [11], which leads us to wonder whether in highly sensitive people it is acting as a protective factor from stress, burnout and compassion fatigue due to susceptibility to their own personal resources [12]. Furthermore, highly sensitive people are characterized by showing higher levels of empathy [10,13,14]. However, unlike what occurs with empathic individuals who show compassion satisfaction [15], their high sensitivity trait could be acting as a risk factor, since in such people, empathy is associated with more suffering from dramatic or problematic situations experienced by others. All of the above, along with prevalence data supplied in studies on high sensitivity, where it has been demonstrated that around 15–20% of the population could have this trait [16], leads us to suggest the need to continue its study. 

At the present time, the situation generated by the COVID-19 pandemic cannot be ignored. In Spain, healthcare workers and educators have had to absorb unexpected changes in their work, not only making it more demanding, but requiring them to adapt to new demands, and work in the context of uncertainty generated by the pandemic, with the imminent urgency of needing to have the resources to help patients/clients. Therefore, the professionals exposed to the effects of this virus have been showing symptoms of stress and burnout [17]. 

In brief, the situation of the healthcare emergency and changes occurring over time are leading to an increase in professional demands, which could generate the appearance of burnout or compassion fatigue [18] or worsen the distress professionals already feel. In addition, as mentioned above, the personal variables of healthcare workers and educators have an important role. Empathy, a characteristic which can be found in highly sensitive individuals [10], as well as in those who show compassion satisfaction in their work [15] should be emphasized.

In this scenario, research advancing knowledge of these syndromes and variables that could be acting as risk or protective factors is required for design of effective intervention strategies adapted to a changing and uncertain context. Recent studies have demonstrated the efficacy of burnout and/or compassion fatigue intervention and prevention programs, showing decrease in burnout and compassion fatigue and increase in compassion satisfaction [19]. Intervention programs have traditionally focused mostly on contextual variables or stressful work conditions [20]. However, in recent years, interest has increased in variables such as compassion satisfaction, personal characteristics, resilience or self-efficacy [4,21]. It has also been observed that training highly sensitive people in intervention programs based on mindfulness, could reduce their stress and increase their sense of personal realization [22]. 

In view of the above, three objectives were posed for this study: (1) to find out the extent to which healthcare and education professionals showed burnout (BO), that is, high scores in emotional exhaustion (EE) and depersonalization (DP) and low in personal accomplishment (PA), and high compassion fatigue (CF) at the beginning of the healthcare emergency caused by the COVID-19 pandemic; (2) to determine whether there were any differences in sensory processing sensitivity (SPS), burnout dimensions, compassion fatigue and compassion satisfaction (CS) by the sector they belonged to; (3) to find out to what extent compassion satisfaction, sensory processing sensitivity and sector influenced their experience of these syndromes as risk and/or protective factors. The final purpose is to advance in designing burnout and compassion fatigue intervention programs for these labor groups.

## 2. Materials and Methods

### 2.1. Participants

The sample came from another study in which 10,821 adults from Spanish-speaking countries (Mexico, Argentina, Colombia, Chile, Peru, Dominican Republic, Costa Rica, Venezuela, Guatemala, Honduras, and others) and countries where the Spanish language is strongly implanted (the United States and Brazil) participated.

The inclusion criteria were Spanish nationality, over 18 years of age and currently employed. 

The exclusion criteria were although from a Spanish-speaking country, not having Spanish nationality, being unemployed, not filling in the data and/or the test battery properly and not giving express informed consent. Thus, following Beaton [23], questionnaires of participants from different cultures and countries, although they spoke the same language, were rejected. Therefore, after applying these criteria, and leaving only participants with Spanish nationality, the sample was reduced to 8358 adults.

**Figure 1:**
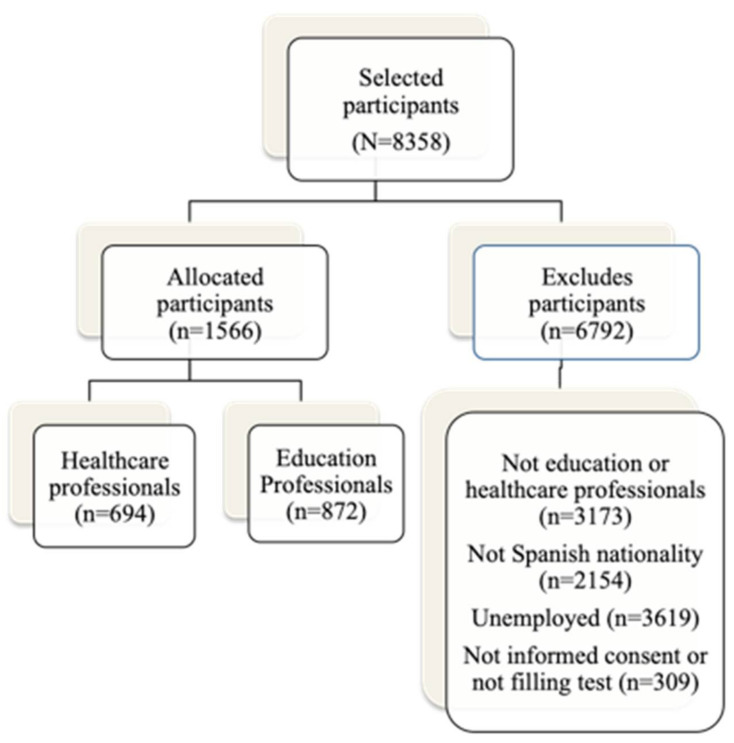
Sample selection procedure.

The sample selection procedure is shown in Figure 1.

From the sample of participants with Spanish nationality, only professionals in the healthcare (n = 694) and education (n = 872) sectors were selected. Therefore, the final sample was made up of 1566 participants aged 22 to 70, of whom 165 were men (10.54%) and 1401 women (89.46%). Of these, 69 men (4.40%) (*mean_age_* = 43.58; *SD* = 11.88) and 625 women (39.91%) (*mean_age_* = 37.79; *SD* = 9.77) were working in healthcare and 96 men (6.14%) (*mean_age_* = 40.04; *SD* = 9.57) and 776 women (49.55%) (*mean_age_* = 37.50; *SD* = 9.62) in education.

The participants were from all of the autonomous regions of Spain, but mostly from Catalonia (20.11%), Madrid (19.41%), Andalusia (14.18%), Valencia (8.56%) and Galicia (6.58%).

### 2.2. Procedure

The study was performed following the code of ethics of the World Medical Association (Helsinki Declaration). All of the participants signed their informed consent. 

Sampling was by convenience, by accessibility. The sample was recruited in a community context. The procedure was: (a) contact the population and associations of highly sensitive people interested in the subject and professionals and workers in Spanish universities, for its diffusion, and (b) fill in the anonymous online tests in 45 to 60 min.

The participants had access to the online application. After reading a brief introduction with the study objectives, they accepted participation and the research conditions. Then they proceeded to the tests which were presented in the same order for all of the participants. Participation was voluntary and received no economic compensation.

The sample was collected in April and May of 2020, coinciding with the beginning of the COVID-19 pandemic, specifically the first two months of confinement derived from the national health emergency.

### 2.3. Measures

An ad hoc questionnaire for sociodemographic and occupational information. This consisted of 12 questions with several answer choices referring to sociodemographic variables (sex, age, marital status, education, number of children, place and autonomous region of residence, occupation, occupation sector, job and time working at present job). 

The highly sensitive person scale (HSPS) [24] by Smolewska [25] adapted to Spanish by Chacón and Pérez-Chacón (2020) [26]. Developed to measure sensory processing sensitivity (SPS), this scale evaluates high sensitivity in adults. The scale consists of 27 items. The items have seven answer choices (0 = totally disagree, 6 = totally agree). This scale has a unidimensional structure with a reliability coefficient of 0.85. In the version by Smolewska et al. [25] three factors were found: ease of excitation, that is, being easily overwhelmed by external and internal stimuli (EOE: items 3, 4, 13, 14, 16, 17, 20, 21, 23, 24, 26 and 27), aesthetic sensitivity, or aesthetic awareness (AES: items 2, 8, 10, 12, 15, 22 and 5), and low sensory threshold, reflecting unpleasant sensory arousal to external stimuli (LST: items 6, 7, 9, 18, 19 and 25), excluding items 1 and 11. The Cronbach’s alpha coefficients were 0.81, 0.72, and 0.78, respectively. To calculate the total SPS score, all the items of the final scale were added. The Cronbach’s alpha coefficients, for this sample, were 0.92. A higher score reflects greater sensory processing sensitivity.

The Spanish Adaptation of the Maslach burnout inventory (MBI) [27,28] was used to evaluate burnout (BO). The scale consists of 22 items and includes three dimensions: emotional exhaustion (EE: items 1, 2, 3, 6, 8, 13, 14, 16, 20), depersonalization (DP: items 5, 10, 11, 15, 22), and decreased personal accomplishment (PA: items 4, 7, 9, 12, 17, 18, 19, 21). The items have seven answer choices (0 = never; 6 = every day). For classification into high, medium, and low burnout, the criteria used were those of the authors applied to the Spanish population sample (*N* = 1.138): EE (low: <15; medium: 15–24; high: >24), DP (low: <4; medium: 4–9; high: >9), and PA (low: <33; medium: 33–39; high: >39). The reliability indices for this sample, according to the Cronbach’s alpha coefficients, were 0.90 (EE), 0.79 (DP), and 0.71 (PA).

The Spanish Adaptation of the Professional Quality of Life Scale (ProQOL-vIV) [29,30] was used to evaluate compassion fatigue and compassion satisfaction. This scale consists of 30 items measuring positive and negative facets of empathy in professionals. The items have six answer choices (0 = never; 5 = always). It includes three dimensions: compassion fatigue (CF: items 2, 5, 7, 9, 11, 13, 14, 23, 25 and 28), compassion satisfaction (CS: items 3, 6, 12, 16, 18, 20, 22, 24, 27 and 30) and burnout (B: items 1, 4, 8, 10, 15, 17, 19, 21, 26 and 29). Items 1, 4, 15, 17 and 29 are scored in reverse. The high, medium and low score classification is: CF (low: ≤7; medium: 8–17; high: ≥18), CS (low: ≤32; medium: 33–41; high: ≥42), and B (low: ≤17; medium: 18–27; high: ≥28). The reliability of the scale was 0.88 and the internal consistency indices were 0.81, 0.84 and 0.07, respectively. This study used the compassion fatigue and compassion satisfaction dimensions. These dimensions are directly related to the objectives of this study and present very good internal consistency values. 

### 2.4. Data Analysis

Statistical analyses were performed with the Statistical Package for the Social Sciences for Windows (SPSS), version 22.0 (SPSS Statistics for Windows, Version 22.0, IBM Corp., Armonk, NY, USA).

To determine the percentage of participants with burnout (BO), the presence of burnout variable was created based on the criteria: EE high >24; DP high >9; PA low <33. Descriptive analyses were done (frequencies, percentages, means and standard deviations) in the different variables for the two groups of professionals, healthcare and educators. Then the presence of high compassion fatigue was determined using the criterion above (CF high: ≥18). The relationship between being a healthcare or an education worker and presence/absence of BO and presence/absence of CF was evaluated using Chi square *(χ*^2^*)*.

The assumption of normality was tested using Kolmogorov–Smirnov and the assumption of homoscedasticity using the Levene statistic. Parametric tests were used in the comparison of means, except for those variables that did not comply with homogeneity of variance.

According to the above, the Student’s *t* and Mann–Whitney (U) were used to test for the existence of significant differences in the scores of MBI (BO dimensions), HSPS (total SPS and its dimensions) and ProQOL-vIV (CF y CS) among healthcare professionals and teachers. The contingency coefficient (*r*^2^*_φ_)* was used to evaluate effect size to complement the Chi square value. 

Similarly, the Cohen’s *d* was calculated using the Lipsey and Wilson [31] formula, considering the sample sizes in the two groups (healthcare/educators workers) and the Student’s *t.* The reference values were <0.30, 0.30–0.50, and >0.50 as small, medium and large sizes, respectively [32].

A Pearson’s *r* correlation analysis was done to find the relationship between BO dimensions, total SPS and its dimensions, CF and CS (MBI, HSPS and ProQOL-vIV, respectively). Effect size values were: small size: <0.30; medium: from 0.30 to 0.49; large: >0.49 [31].

Then, to find out the weight of SPS and each dimension as well as CS in each BO dimensions and in CF, a multiple linear regression analysis was performed using the “enter” method.

## 3. Results

### 3.1. Preliminary Analysis

The groups were equivalent in sex (*p* = 0.495), age (*p* = 0.247), marital status (*p* = 0.198) and number of children (*p* = 0.412). Differences were found in education (*p* = 0.000) and type of professional activity (*p* = 0.000). Sample characteristics are presented in Table 1.

### 3.2. Presence of Burnout and Compassion Fatigue

The relationship between healthcare worker and educator and presence/absence of BO and high CF was evaluated using Chi square (*χ*^2^). The frequency and percentage analysis used crossed tables between presence of BO and high CF by professional sector 4.3% (*n* = 30) of the healthcare workers fulfilled criteria for presence of BO compared to 4.2% of educators, with non-significant *χ*^2^. However, the percentages of high CF increased noticeably in both professional sectors, surpassing the criterion by a higher percentage in 71.4% (*n* = 623) of educators compared to 65.1% of healthcare professionals. Significant associations were found in CF (*χ*^2^ = 7.161; *p* = 0.007; *r*^2^*_φ_* = 0.067).

### 3.3. Comparison of Sensory Processing Sensitivity, Burnout Dimensions, Compassion Fatigue and Compassion Satisfaction

The mean scores, Student’s t-test and Mann–Whitney for comparison of means are shown in Table 2. Statistically significant differences were observed in high sensitivity (SPS) and its dimensions (EOE, LST and AES), where the means were highest for educators, in both total score and dimensions. Significant differences were observed in the PA and DP dimensions, with healthcare professionals scoring higher in both dimensions. Finally, significant differences in CF were found, where educators had higher mean scores, although it should be considered that the effect sizes were small (see Table 2).

### 3.4. Relationship between Sensory Processing Sensitivity, Burnout Dimensions, Compassion Fatigue and Compassion Satisfaction

Regarding the relationship between total SPS and the BO dimensions (EE, DP, PA), small variations were found between sectors. Both in healthcare workers and educators, the relationships with EE were significant (*r* = 0.20, *p* = 0.000 and *r* = 0.28, *p* = 0.000, respectively), but not with PA (*p* = 0.635 and *p* = 0.800, respectively). The relationship between DP and high sensitivity was significant in the group of educators (*r* = 0.07, *p* = 0.023) (see Table 3).

The relationships between BO (EE, DP and PA) and SPS (EOE, LST, AES) dimensions, were direct and significant between EE and EOE and AES in both healthcare and education sectors, but not with LST (EOE: *r* = 0.22, *p* = 0.000 and *r* = 0.31, *p* = 0.000; LST: *r* = 0.09, *p* = 0.018 and *r* = 0.15, *p* = 0.000; AES: *r* = 0.14, *p* = 0.000 and *r* = 0.21, *p* = 0.000; respectively). Similarly, the relationships established between the high sensitivity subscales with DP and PA were different in both sectors. Specifically, there was a direct significant relationship between EOE and DP in the group of educators (*r* = 0.09, *p* = 0.004). The PA dimension was significantly directly related to LST (*r* = 0.17, *p* = 0.000 and *r* = 0.13, *p* = 0.000, respectively) in both sectors, and the opposite with EOE in educators (*r* = −0.07, *p* = 0.023) with small effect sizes.

CF and total high sensitivity (SPS) (*r* = 0.37, *p* = 0.000 and *r* = 0.38, *p* = 0.000) and their dimensions were directly and significantly related in both the healthcare and education sectors (EOE: *r* = 0.38, *p* = 0.000 and *r* = 0.37, *p* = 0.000; LST: *r* = 0.23, *p* = 0.000 and *r* = 0.23, *p* = 0.000; AES: *r* = 0.31, *p* = 0.000 and *r* = 0.34, *p* = 0.000; respectively). 

With respect to CS, significant inverse relationships were found in healthcare and education professionals with CF (*r* = −0.22, *p* = 0.000 and *r* = −0.10, *p* = 0.002; respectively) and the EE and DP dimensions (EE: *r* = −0.53, *p* = 0.000 and *r* = −0.44, *p* = 0.000; DP: *r* = −0.40, *p* = 0.000 and *r* = −0.34, *p* = 0.000; respectively). However, with PA the relationship was direct (*r* = 0.66, *p* = 0.000 and *r* = 0.62, *p* = 0.000; respectively).

Lastly, there were no significant relationships between CS and total SPS in either healthcare workers (*p* = 0.334) or educators (*p* = 0.452). A significant inverse relationship was found between the high sensitivity dimensions (SPS) and EOE (*r* = −0.09, *p* = 0.014) in healthcare workers and direct with LST (*r* = 0.10, *p* = 0.002) in educators, with low effect sizes.

### 3.5. Influence of High Sensitivity and Compassion Satisfaction on Burnout Dimensions and Compassion Fatigue

Linear regression analyses were done to determine the weight of high sensitivity, CS and sector on BO dimensions, as well as on CF. Four regression analyses were performed with four criterion variables, the three BO dimensions (EE, PA and DP) and CF. The predictor variables were SPS dimensions (EOE, LST and AES), CS and sector (healthcare or education). In each analysis, the dimensions of high sensitivity (SPS) which had previously been shown to have significant correlations with the criterion variables were included. The total SPS variable had to be excluded from the analyses because it caused collinearity. The “enter” method was used for the analyses. 

Table 4 shows the four models. All of them were significant, but more variance was explained in the PA and EE models.

The predictor variables in the model estimated for EE were the SPS dimensions (EOE, AES, LST), CS and sector. The resulting model explained 30.1% (*R*^2^ = 0.301) of EE scores (*F* = 134.173; *p* = 0.000), again resulting in significant effects of CS and EOE. As in the previous model, CS had a large effect size. It affected EE negatively, while EOE had a positive effect. 

The EOE and LST dimensions, CS and the sector variable were entered for PA. The resulting model explained 43.8% of the variance (*R*^2^ = 0.438) (*F* = 304.213; *p* = 0.000). All the effects were significant, and the largest effect size was the sector variable (*B* = −1.42), which was negative, as was the EOE variable. Therefore, belonging to the healthcare sector and scoring high on EOE reduced PA. However, CS and the LST scores contributed to increasing PA. 

In the last BO dimension analyzed (DP), the variables EOE, CS and sector were entered in the model as the predictor variables. The model estimated explained 15.3% (*R*^2^ = 0.153) of the variance (*F* = 95.046; *p* = 0.000). The regression coefficients were significant for CS and sector, both with negative effect sizes, which demonstrates that high scores in CS and being a healthcare professional would contribute to reducing DP. 

In the model estimated for CF, the EOE, LST and AES dimensions, CS and sector were entered as predictor variables. The resulting model explained 17.3% (*R*^2^ = 0.173) of the variance (*F* = 67.278; *p* = 0.000). Only the effects of EOE and AES were significant, with positive coefficients, as well as CS with a negative effect size. Thus, it could be said that regardless of sector, while high scores in EOE and AES would increase CF, scores in CS would reduce them. 

## 4. Discussion

The first objective posed in this study was to find out the extent to which healthcare and education professionals suffered from burnout (high emotional exhaustion and depersonalization and Low personal accomplishment) and high compassion fatigue in the period at the start of the COVID-19 healthcare emergency. 

The results found show that prevalence of burnout was around 4% in both sectors, and no significant association was found between burnout and sector. These data are lower than those reported in previous studies on the burnout syndrome in healthcare and education, which showed prevalence of about 17–20% [33]. However existing studies with prevalence data are few, and in our study the criteria used were very restrictive, exclusively selecting those participants with high scores in emotional exhaustion and depersonalization and low in personal accomplishment. In fact, mean scores for emotional exhaustion and depersonalization dimensions are higher than those reported in studies [33,34].

The percentages of high presence of compassion fatigue increased considerably in both sectors, and were higher in education than in healthcare. Unlike burnout, a significant association was found between high compassion fatigue and sector. These findings are higher than the results found in previous studies [15,35], which may be explained by the coincidence of having collected the data at the beginning of the healthcare emergency derived from the pandemic. The uncertainty of COVID-19 development in the context of the healthcare emergency has led to an increase in work overload and emotional burden in both sectors. Furthermore, the fact that the percentage of professionals with high compassion fatigue is greater in the teaching sector than in healthcare, although contrary to what might be expected [35], is not surprising either. The moment in the academic year when various teaching tools were urgently started up to continue with the objectives of the last period of classes meant that teachers not only had to adapt to working remotely, but often had to attend to personal problems of their students and families, which might not have occurred in another situation. This, along with the lack of resources for dealing with these new demands, doubtless worsened compassion fatigue.

The second objective was to find out the differences in the variables analyzed between the healthcare and education sectors. In view of the mean scores in burnout dimensions, it may be said that both sectors scored high in emotional exhaustion, and the only significant differences were in depersonalization and personal realization. In this case, the means were higher in the healthcare sector than in education. In both sectors, the scores in depersonalization were moderate, while in personal realization, the score in the healthcare sector could be considered high and in education moderate. 

These findings are congruent with previous studies [1], and could, in turn, explain the results found for the more frequent high compassion fatigue in teachers. Healthcare professionals, along with other coping strategies, use a certain distancing similar to depersonalization to manage variability in their experiences with people who are suffering. In this case, depersonalization would protect them from the negative effect that empathizing with the problems of the people they care for could have. In spite of this, personal realization experienced in their work is higher. It should be recalled that during the months when the data were collected, Spain was confined by the healthcare state of emergency. During those months, healthcare workers were considered heroes by most of society. That is, they had the emotional support of a very large number of people who valued and recognized their work. On the contrary, the action of teachers was often severely criticized, as students and their families were going through this situation of uncertainty with heavy overload and stress, frequently attributing the fault to these professionals. In fact, in view of the mean scores on compassion fatigue, just as with emotional exhaustion, mean scores in both sectors were very high, but in this case, significant differences were observed between the two sectors, where the mean score was higher in education than in healthcare. 

Although, sensory processing sensitivity and compassion satisfaction were usually observed to be higher in education, that compassion satisfaction was moderate in both sectors and the differences were only significant in sensorial processing sensitivity. Contrary to compassion fatigue, in education, difficulties and obstacles to teaching and education in general, are usually rewarded with initiatives based on creativity, experimentation, contact with nature, linguistic expression and dramatization, etc., carried out with the students, and in highly sensitive people, these qualities are very gratifying. However, during recent months, these experiences have been reduced and could especially affect compassion satisfaction [11], whereas in the healthcare sector, as mentioned, the recognition and human and professional dedication contributed in large part to compassion satisfaction, even more so in highly sensitive people whose own trait qualities impact directly on the search for wellbeing of others who are suffering. This in turn, could also explain our results, and leads us to believe in the importance of working on adequate management of empathy in both sectors, especially education. 

It should be mentioned that the effect sizes of these differences were very small, so they should be taken with caution in generalizing the differences found between sectors in this sample to their respective reference populations. A possible explanation could focus on two points. On one hand, the difference in scores is minimal, and on the other, that these differences are given by the exceptional nature of the situation in which the data were collected, a situation of severe stress as a consequence of the healthcare emergency [18]. 

The third objective posed was to find out to what extent compassion satisfaction, sensory processing sensitivity and sector influence burnout dimensions and compassion fatigue, whether acting as risk and/or protective factors. 

From the results found, emotional exhaustion was observed to be reduced in both sectors with increased compassion satisfaction, while the increase in ease of excitation would contribute to increasing emotional exhaustion, with compassion satisfaction having a larger effect size. In agreement with these results, some authors [36] have found that ease of excitation predicts the reaction to stress, whereas others [8] have found that higher compassion satisfaction could predict less emotional exhaustion. 

Sector was significant in the other two dimensions of burnout, personal realization and depersonalization. Being a healthcare professional was a risk factor for personal realization and as a protective factor from appearance of depersonalization. In personal realization, belonging to the healthcare sector and scoring high on ease of excitation reduced the scores, and acted as a risk factor. However, the increase in scores in compassion satisfaction and low sensorial threshold contributed to increasing personal realization, and these variables therefore acted as personal realization protective factors. Similarly, high scores in compassion satisfaction and pertaining to the healthcare sector contributed to reducing depersonalization. 

In personal realization, the findings that compassion satisfaction along with low sensorial threshold acted as protective factor, show the two sides of high sensitivity. When high sensitivity is managed adequately, it can become a personal resource with strong potential for generating personal realization in one’s work. In other words, higher sensory susceptibility to any change in the work environment could lead to selecting coping strategies more adequate to the type of demand (emotion or problem) to which one is exposed [37,38]. Likewise, being a healthcare professional and feeling overburdened by internal or external demands, would become a risk factor. This attribute makes sense, because the overburden itself is considered a psychosocial risk factor and healthcare professionals, by being exposed to high emotional demands, become an at-risk group [39].

In addition, pertaining to the healthcare sector and showing high compassion satisfaction are protective factors from developing depersonalization. The importance of promoting compassion satisfaction among these professionals as a means of promoting wellbeing is therefore be inferred from this finding [1]. 

Finally, in view of the influence of compassion satisfaction and high sensitivity on compassion fatigue observed, regardless of sector, the dimensions of sensory processing sensitivity, ease of excitation and aesthetic sensitivity, increased compassion fatigue, while the effect of compassion satisfaction reduced compassion fatigue. These findings enable us to confirm that sensory processing sensitivity is a factor influencing quality of life of care professionals [36]. Our results report on a high percentage of professionals with compassion fatigue, predicted by ease of excitation and aesthetic sensitivity. Beyond the effect of the dimensions of high sensitivity, as a protective factor, it was found that compassion satisfaction contributed to reducing compassion fatigue. 

In brief, the findings of this study demonstrate the presence of burnout and high compassion fatigue in healthcare and education professionals, displaying compassion fatigue as an emerging psychosocial risk in education which was made more severe under the conditions of study, that is at the beginning of the COVID-19 pandemic. The weight of sensory processing sensitivity and compassion satisfaction as risk or protective factors is also evident. Therefore, it is recommended that prevention programs include strategies for adequate management of sensitivity, empathy and feeling compassion, in line with what has been discussed in previous studies [4,15,21,22] for the wellbeing of these professionals in their professional practice.

## 5. Conclusions

The objectives of this study showed the existence of burnout and compassion fatigue among healthcare and education professionals in the sample of Spanish citizens studied. More personal realization and depersonalization was observed in healthcare workers and higher compassion fatigue in educators. Moreover, certain characteristics of sensory processing sensitivity facilitated the presence of burnout and compassion fatigue. The characteristics of high sensitivity acted as risk factors for burnout dimensions and compassion fatigue, except for low sensory threshold, which acted as a protective factor for personal realization. On the contrary, compassion satisfaction was a protective factor from burnout dimensions and compassion fatigue. As to sector, being a healthcare professional became a risk factor for personal realization, but was also protection, as it diminished depersonalization. We believe, because healthcare workers and educators are at-risk labor groups for developing burnout in the course of their social service work, that the results of this study could have been worsened by the situation in each of these sectors during the time data were collected, which coincided with the beginning of the state of emergency due to COVID-19. Nevertheless, we believe that regardless of the effects of the pandemic, healthcare professionals and educators would be labor groups which could benefit in their respective professions form interventions directed at managing empathy satisfactorily to prevent the appearance of burnout and compassion fatigue, especially in highly sensitive person.

## 6. Limitations

As possible limitations of this study, those specifics of online evaluation should be mentioned, among them, the impossibility of controlling certain strange variables that could have interfered in completing the questionnaires. Furthermore, having performed the evaluation at the beginning of the pandemic, some professionals may have been experiencing severe stress, which could have biased their answers. Without doubt, this study should be completed with a second evaluation to follow up on these professionals. This would enable us to determine with greater accuracy whether the results found here are the direct consequence of the time the evaluation was made, the first months of the state of emergency, or if on the contrary, they are maintained. In fact, the authors are planning to repeat the evaluation next year to continue this line of research and clarify the questions associated with prognosis and evolution. 

## Figures and Tables

**Table 1:** Sample characteristics.

	*N* = 1566		Type of Professional Activity				
			Healthcare (*n* = 694)		Education (*n* = 872)		*t*/*X*^2^
	*F*	*%*	*F*	*%*	*F*	*%*	
Sex							
Men	165	10.54	69	4.40	96	6.14	*t*_(1563)_ = 1.159 *p* = 0.247
Women	1401	89.46	625	39.91	776	49.55	
Age							
<30	407	25.99	171	10.92	236	15.07	
31–40	551	35.18	254	16.22	297	18.96	*X*^2^_(4)_ = 12.667 *p* = 0.013 *
41–50	413	26.37	165	10.54	248	15.83	
51–60	163	10.41	82	5.24	81	5.17	
>61	25	1.59	17	1.08	8	0.51	
Unspecified	7	0.45	5	0.32	2	0.12	
Education							
Elementary School	4	0.25	2	0.12	2	0.12	
Middle School	14	0.89	9	0.57	5	0.32	*X*^2^_(5)_ = 54.620 *p* = 0.000 **
High School	216	13.08	125	7.98	91	5.82	
Graduate	688	43.93	285	18.20	403	25.73	
Postgraduate	516	32.95	236	15.07	280	17.88	
Doctorate	128	8.17	37	2.36	91	5.81	
Marital status							
Single	504	32.18	214	13.66	290	18.52	
Married	477	30.46	224	14.30	253	16.16	*X*^2^_(6)_ = 9.832 *p* = 0.198
Living together	377	24.07	167	10.66	210	13.40	
Widow/widower	9	0.57	3	0.19	6	0.38	
Separated marriage	45	2.87	18	1.15	27	1.72	
Divorced	103	6.58	52	3.32	51	3.26	
Others	51	3.26	16	1.02	35	2.24	
Number of children							
0	901	57.54	394	25.16	507	32.38	*X*^2^_(5)_ = 6.097 *p* = 0.412
1	235	15.00	106	6.76	129	8.24	
2	303	19.35	139	8.88	164	10.47	
3	55	3.51	28	1.79	27	1.72	
>3	15	0.96	4	0.25	11	0.71	
Unspecified	57	3.64	23	1.47	34	2.17	
Occupation							
Outside company	1237	79.00	502	32.05	735	46.93	*X*^2^_(1)_ = 33.280 *p* = 0.000 **
Autonomous company	329	21.00	192	12.27	137	8.75	

** *p* ≤ 0.01, * *p* ≤ 0.05.

**Table 2:** Comparisons between groups.

Variables	Healthcare		Education				
					Comparison of Means		
	*M*	*SD*	*M*	*SD*	*t/U*	*Sign*	*d Cohen*
Sensory processing sensitivity (Total SPS)	125.86	23.25	130.35	20.99	*U* = 341,082	0.000 **	−0.20
Ease of excitation (EOE)	54.97	10.77	57.10	10.30	*t* = −3.999	0.000 **	−0.20
Low sensory threshold (LST)	34.11	6.42	35.11	5.76	*U* = 332,987.50	0.001 **	−0.16
Aesthetic sensitivity (AES)	33.17	6.88	34.35	6.23	*U* = 331,237	0.000 **	−0.18
Emotional exhaustion (EE)	26.92	12.81	27.45	12.12	*U* = 309,532.50	0.434	−0.04
Personal accomplishment (PA)	39.93	6.84	38.91	7.01	*t* = 2.880	0.004 **	0.15
Depersonalization (DP)	5.97	5.45	4.91	4.83	*U* = 270,807.50	0.000 **	0.21
Compassion fatigue (CF)	21.16	7.95	22.20	7.78	*t* = −2.597	0.010 *	−0.13
Compassion satisfaction (CS)	37.41	7.69	38.12	7.24	*t* = −1.859	0.063	−0.09

** *p* ≤ 0.01, * *p* ≤ 0.05.

**Table 3:** Relationship between healthcare and education variables.

Healthcare (*n* = 694)										Education (*n* = 872)									
				HSPS		MBI			ProQO-vIV					HSPS		MBI			ProQOL-vIV
		Total SPS	EOE	LST	AES	EE	DP	PA	CF			Total SPS	EOE	LST	AES	EE	DP	PA	CF
^(a)^ Sensory processing sensitivity (HSPS)	Total SPS	1								Sensory processing sensitivity (HSPS)	Total SPS	1							
	EOE	0.91 ** 0.000	1 0.000								EOE	0.91 ** 0.000	1						
	LST	0.84 ** 0.000	0.63 ** 0.000	1 0.000							LST	0.79 ** 0.000	0.56 ** 0.000	1					
	AES	0.87 ** 0.000	0.67 ** 0.000	0.68 ** 0.000	1 0.000						AES	0.86 ** 0.000	0.67 ** 0.000	0.60 ** 0.000	1				
^(b)^ Burnout (MBI)	EE	0.20 ** 0.000	0.22 ** 0.000	0.09 * 0018	0.14 ** 0.000	1				Burnout (MBI)	EE	0.28 ** 0.000	0.31 ** 0.000	0.15 ** 0.000	0.21 ** 0.000	1			
	DP	0.01 0.744	0.058 0.128	−0.05 0.156	0.00 0.901	0.40 ** 0.000	1				DP	0.07 * 0.023	0.09 ** 0.004	0.03 0.347	0.03 0.332	0.41 ** 0.000	1		
	PA	0.01 0.635	−0.06 0.088	0.17 ** 0.000	0.02 0.529	−0.39 ** 0.000	−0.31 ** 0.000	1			PA	0.00 0.800	−0.07 * 0.025	0.13 ** 0.000	0.02 0.458	−0.31 ** 0.000	−0.32 ** 0.000	1	
^(c)^ Quality of life (ProQOL-vIV)	CF	0.37 ** 0.000	0.38 ** 0.000	0.23 ** 0.000	0.31 ** 0.000	0.56 ** 0.000	0.24 ** 0.000	−0.23 ** 0.000	1	Quality of life (ProQOL−vIV)	CF	0.38 ** 0.000	0.37 ** 0.000	0.23 ** 0.000	0.34 ** 0.000	0.56 ** 0.000	0.31 ** 0.000	−0.16 ** 0.000	1
	CS	−0.03 0.334	−0.09 * 0.014	0.07 0.070	−0.01 0.810	−0.53 ** 0.000	−0.40 ** 0.000	0.66 ** 0.000	−0.22 ** 0.000		CS	0.02 0.452	−0.03 0.445	0.10 ** 0.002	0.03 0.331	−0.44 ** 0.000	−0.34 ** 0.000	0.62 ** 0.000	−0.10 ** 0.000

** *p* ≤ 0.01, * *p* ≤ 0.05 Note: effect size: small magnitude ratio: <0.30; mean: between 0.30 y 0.49; high: >0.49 ^(a)^ Sensory processing sensitivity (SPS) (EOE: ease of excitation, LST: low sensory threshold, AES: aesthetic sensitivity), ^(b)^ dimensions of burnout syndrome (BO) (EE: emotional exhaustion, DP: depersonalization, PA personal accomplishment), ^(c)^ quality of life (ProQOL-vIV) (CF: compassion fatigue, CS: compassion satisfaction).

**Table 4:** Regression analyses. Influence of high sensitivity and compassion satisfaction on burnout dimensions and compassion fatigue.

	*Beta*	*Standard Error*	*t*	*p*
Emotional exhaustion (EE)				
*R*^2^ = 0.301				
(Constant)	35.774	2.438	14.671	0.000 **
Compassion satisfaction (CS)	−0.795	0.036	−22.190	0.000 **
Ease of excitation (EOE)	0.260	0.036	7.238	0.000 **
Personal accomplishment (PA)				
*R*^2^ = 0.438				
(Constant)	17.574	1.220	14.401	0.000 **
Compassion satisfaction (CS)	0.575	0.018	31.998	0.000 **
Sector	−1.429	0.267	−5.345	0.000 **
Ease of excitation (EOE)	−0.098	0.016	−6.135	0.000 **
Low sensory threshold (LST)	0.214	0.027	7.802	0.000 **
Depersonalization (DP)				
*R*^2^ = 0.153				
(Constant)	14.647	1.053	13.912	0.000 **
Compassion satisfaction (CS)	−0.257	0.016	−15.951	0.000 **
Sector	−0.939	0.242	−3.873	0.000 **
Compassion fatigue (CF)				
*R*^2^ = 0.173				
(Constant)	6.934	1.675	4.140	0.000 **
Compassion satisfaction (CS)	−0.148	0.025	−6.030	0.000 **
Ease of excitation (EOE)	0.206	0.025	8.350	0.000 **
Aesthetic sensitivity (AES)	0.187	0.041	4.569	0.000 **

** *p* ≤ 0.01.

## Data Availability

The data presented in this article are available on request from the corresponding author.
